# COVID-19 impacts on household energy & food security in a Kenyan informal settlement: The need for integrated approaches to the SDGs

**DOI:** 10.1016/j.rser.2021.111018

**Published:** 2021-07

**Authors:** Matthew Shupler, James Mwitari, Arthur Gohole, Rachel Anderson de Cuevas, Elisa Puzzolo, Iva Čukić, Emily Nix, Daniel Pope

**Affiliations:** a Department of Public Health, Policy and Systems, University of Liverpool, Liverpool, United Kingdom; b School of Public Health, Amref International University, Nairobi, Kenya; c Global LPG Partnership (GLPGP), 654 Madison Avenue, New York, United States

**Keywords:** Clean cooking fuels, Liquefied petroleum gas, COVID-19, Food security, Energy security, Pay-as-you-go, Informal urban settlement

## Abstract

This longitudinal study presents the joint effects of a COVID-19 community lockdown on household energy and food security in an informal settlement in Nairobi, Kenya. Randomly administered surveys were completed from December 2019–March 2020 before community lockdown (n = 474) and repeated in April 2020 during lockdown (n = 194). Nearly universal (95%) income decline occurred during the lockdown and led to 88% of households reporting food insecurity. During lockdown, a quarter of households (n = 17) using liquefied petroleum gas (LPG), a cleaner cooking fuel typically available in pre-set quantities (e.g. 6 kg cylinders), switched to polluting cooking fuels (kerosene, wood), which could be purchased in smaller amounts or gathered for free. Household size increases during lockdown also led to participants’ altering their cooking fuel, and changing their cooking behaviors and foods consumed. Further, households more likely to switch away from LPG had lower consumption prior to lockdown and had suffered greater income loss, compared with households that continued to use LPG. Thus, inequities in clean cooking fuel access may have been exacerbated by COVID-19 lockdown. These findings demonstrate the complex relationship between household demographics, financial strain, diet and cooking patterns, and present the opportunity for a food-energy nexus approach to address multiple Sustainable Development Goals (SDGs): achieving zero hunger (SDG 2) and universal affordable, modern and clean energy access (SDG 7) by 2030. Ensuring that LPG is affordable, accessible and meets the dietary and cooking needs of families should be a policy priority for helping improve food and energy security among the urban poor.

## Highlights

• One quarter of informal urban settlement households using LPG switched their cooking fuel to wood or kerosene during COVID-19 lockdown.
• Nine in ten households reported income decline/cessation and food insecurity as a result of lockdown.
• Dietary changes were 40% more prevalent among households switching their cooking fuel during lockdown (85%) than those not (46%).
• Increases in cooking fuel prices and changes in cooking frequency and dietary patterns were observed during lockdown.
• Inequalities in clean cooking access may be deepened by the COVID-19 pandemic.

## Graphical abstract

**Figure:**
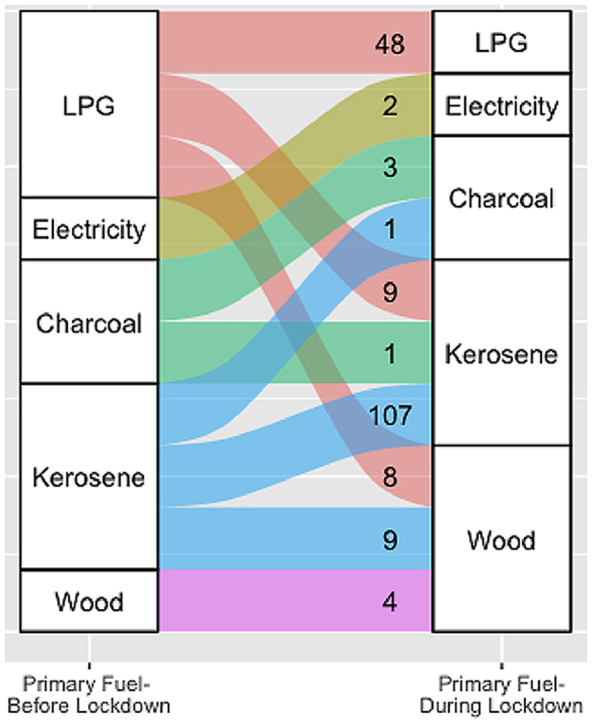

## Abbreviations

HAP: Household air pollution
LMICs: Low- and middle-income countries
Ksh: Kenyan Shilling
LPG: Liquefied petroleum gas
PAYG: Pay-as-you-go
PM_2.5_: Particulate matter less than 2.5 μm
SDG: Sustainable Development Goal
WHO: World Health Organization

## 1 Introduction

Approximately 3.8 billion people (over 40% of the global population), primarily in low- and middle-income countries in Africa, Asia and South America, lack access to clean, modern and affordable sources of household energy [1]. These households generally depend on polluting fuels, including biomass (e.g. wood, charcoal), coal and kerosene, for cooking, heating and lighting, which negatively affects their health, livelihoods and the climate [[2], [3], [4]]. The burning of polluting fuels exposes individuals to unsafe levels of fine particulate matter (PM_2.5_) from household air pollution [5], which makes them susceptible to various infectious (e.g. tuberculosis, pneumonia) [[6], [7], [8], [9]] and non-communicable respiratory (e.g. lung cancer, chronic obstructive pulmonary disease) and cardiovascular diseases (e.g. stroke, heart disease) [[10], [11], [12], [13], [14], [15], [16]]. The Global Burden of Disease (GBD) study attributed a total of 3.6 million deaths annually from exposure to household air pollution (HAP) in 2019, making it the 2nd highest environmental risk factor [17].

Combustion of polluting fuels emits long-lived (e.g. CO_2_) and short-lived (e.g. black carbon) climate-forcing pollutants [4]. Black carbon, which consists of the dark component of particulate matter, has strong visible light absorption properties [[18], [19], [20]] and is estimated to have the second largest radiative forcing, following only CO_2_ [19,21]. Combustion of biomass fuels for household energy is estimated to contribute to between one-third and a half of all global anthropogenic emissions of black carbon [22,23]. Unsustainable harvesting of wood for cooking and charcoal production can cause deforestation, contributing to increased atmospheric CO_2_ and leading to loss of biodiversity [4,24]. As women and young girls traditionally assume the role as the primary cook of the household in low-income settings, they may spend several hours a week collecting and transporting biomass fuels [[25], [26], [27], [28]]. The resulting ‘time poverty’ can exacerbate gender inequality [[29], [30], [31]].

Household energy shortages have also been shown to impact food security in developing economies in the long term [32,33]. A report by the World Food Programme states that high fuel costs and inadequate access to energy requires households to employ coping strategies including trading food rations for cooking fuel and skipping or undercooking food [34]. Fuelwood scarcity also contributes to households preparing food with low nutritional value requiring less cooking time and not sufficiently boiling water to eliminate impurities [35].

While the number of individuals with access to adequate food and clean household energy sources has increased over the last decade, population growth has exceeded gains in access [1,36]. Therefore, achieving the United Nation's Sustainable Development Goals (SDG) 2 (zero hunger) and SDG 7 (universal access to clean, modern and affordable household energy) by 2030 is unlikely [[37], [38], [39]]. Food insecurity rose by an estimated 60 million between 2014 and 2019 [40] and use of polluting cooking fuels has stagnated at around three billion people since 2010 [41]. It is currently estimated that nearly 30% percent of people worldwide will rely on polluting fuels for cooking in 2030 [42]. Given these challenges, a food-energy nexus framework [33,38] can simultaneously help reduce food insecurity, promote access to modern household energy and decrease the disproportionate health burden faced by women and children [3].

Sub-Saharan Africa (SSA), one of the regions suffering the highest burden of energy and food insecurity, will be highly influential in determining global energy and food security trends over the coming decades [43]. Over 85% of the population in SSA cooked with polluting fuels in 2018, twice the global average, and 70% of the global population without access to electricity lived in SSA in 2018 [42]. The total number of people using polluting fuels for household energy in SSA rose from 750 million in 2010 to 890 million in 2018 [36]. In 2016, a quarter of individuals in SSA were estimated to be food insecure [44].

Reaching universal food and energy security in SSA by 2030 will be more challenging with the onset of the coronavirus disease 19 (COVID-19) pandemic in 2020. Some of the immediate economic impacts of COVID-19 in SSA included income loss, particularly among informal sector workers who typically live on daily wages, a decline in income from remittances, and food system disruptions [45]. A recent World Bank report estimates that an additional 26 to 40 million sub-Saharan Africans could fall into poverty and suffer from food insecurity and hunger due to the COVID-19 pandemic [46]. The International Energy Agency (IEA) World Energy Outlook 2020 Report estimates that the rise in poverty levels has made electricity unaffordable for over 13 million people in SAA with electricity connections, representing a 2% drop in electrified households in 2020 2020 [36]. A decrease in the number of households with access to clean energy for cooking due to COVID-19 is also expected [47].

Informal urban settlements, which are home to over 50% of the urban SSA population [48], present a high risk of COVID-19 transmission due to high population density, inadequate housing and limited water and sanitation facilities [49]. As a result, national lockdowns implemented by SSA governments at the onset of the COVID-19 pandemic were enforced in informal urban settlements [50,51]. However, residents were unable to comply with many of the public health guidelines in cramped spaces, including maintaining social distancing [52,53]. Livelihoods were negatively impacted as many working in the informal sector and relying on daily income-generating activities to support their families were unable to work [54]; female informal sector workers were among the first to lose their jobs [53]. Many families experienced increased food insecurity due to the mandatory lockdowns [55].

This paper investigates the drivers of urban food and energy insecurity in the context of the COVID-19 pandemic in an informal settlement in Nairobi, Kenya. Nairobi has one of the largest populations living in informal urban settlements in SSA; over two million people live in approximately 175 informal settlements, making up 56% of Nairobi's population yet occupying only 6% of the land [56,57]. A previous study conducted in multiple informal settlements in Nairobi found that 86% of residents suffered total or partial loss of income due to COVID-19 lockdown, and 74% reported eating less or skipping meals due to insufficient income [58]. Building on these findings, this study explores the relationship between food and energy security during lockdown and investigates opportunities for innovative technological solutions to mitigate the negative impacts of COVID-19 lockdown on household energy among the urban poor. These learnings can help regain momentum towards achieving SDG 2 and SDG 7 b y 2030 [59].

This paper is organized into several sections. Section 2 highlights prior research on food and energy insecurity in informal urban settlements and the potential for interim clean household energy solutions in SSA. Section 3 introduces the data source and methodology used in this study. Section 4 summarizes and discusses the empirical results. Section 5 presents conclusions and policy recommendations.

## 2 Food-energy nexus in Nairobi's informal settlements

### 2.1 The food environment

Nearly 30% of the Kenyan population were undernourished in the period between 2016 and 2018 [45]. It is estimated that 85% of those living in informal urban settlements in Nairobi were food insecure prior to the COVID-19 pandemic [60,61]. A previous study found that 40% of children living in informal settlements in Nairobi were stunted and 10% were underweight [62]. Although agriculture is a key food source and income generator in rural Kenyan communities [63], in urban areas food is more commonly purchased from small-scale owner-operated businesses, including street vendors and hawkers, where many female workers are employed [57,64]. Some food vendors sell ready meals directly in the community that are consumed by households that cannot afford to prepare food from raw ingredients and cooking fuel prices [65].

Given the complexity of the food environment in informal settlements, reasons for shifts in the quantity, quality and safety of food eaten can be multifactorial, including proximity to markets, time constraints and access to cooking fuels [66]. An examination of how fuel prices and access to clean cooking can impact dietary behavior in an informal settlement may therefore help determine some causes of food insecurity.

### 2.2 Clean energy access

Kenya is one of several African countries that has set aspirational targets for rapid market expansion of liquefied petroleum gas (LPG) as a cleaner fuel for cooking [67,68]. LPG burns very efficiently and offers a scalable, intermediate solution to clean cooking in SSA due to lower infrastructure requirements [69]. LPG is currently used for cooking by over 2.5 billion people [70], and has experienced rapid expansion in Latin America [71], India [72] and Indonesia [73]. Despite being a fossil fuel, LPG emits no black carbon and low levels of PM_2.5_ [74,75]. LPG use can have a neutral or cooling effect on climate when accounting for the reduction in emissions of CO_2_ and black carbon from replacement of biomass combustion [24,76], and reductions in localized deforestation [69,77].

Studies have found that LPG can save the primary cook, typically women, an average of 40–45 min of cooking time per day [27,28], and prevent the need to gather firewood, which can take up to an hour or more per day in some cases [35]. However, despite the potential time savings and health benefits of using LPG, many primary cooks still prefer to use biomass as their primary cooking fuel. One reason that firewood or charcoal may be considered preferable to LPG is because of the incompatibility of LPG with certain local cooking practices [78,79]. For example, primary cooks may think that certain meals may taste unpleasant when prepared with LPG, are concerned about using LPG for long-cooking duration foods and are unable to cook sizeable meals due to smaller LPG stove burners that cannot accommodate large pots [80,81].

A qualitative study conducted in Kibera, an informal settlement in Nairobi, found that kerosene was deemed unsuitable for grilling certain meats and that using an electric stove would cause chicken or chapatti to “look and taste bad” [82]. Other participants in the study said that ugali, a Kenyan staple food that is a porridge consisting of maize flour and water, does not taste good when cooked on a kerosene stove; the individuals would therefore not switch to kerosene even if it was affordable. These findings demonstrate the links between the cooking fuel used and a family's dietary choices [39], highlighting the need to consider a food-energy nexus.

### 2.3 Potential impacts of COVID-19 on household energy decisions

Household energy usage is known to be variable in low-income settings due to unexpected or seasonal changes in income [72,83], and cooking fuels are routinely ‘stacked’ (use of clean fuels alongside polluting fuels) [84,85] to meet all household cooking needs. The study from the Kibera informal settlement showed that participants believed that supply of LPG was unreliable and the fuel was too expensive to use for all cooking tasks [82].

While LPG must usually be purchased in pre-set, fixed quantities (e.g. 6 kg cylinders), polluting fuels can usually be purchased in small, daily amounts (kerosene) or gathered for free (wood) [83,86]. With household energy (20%) and food (40%) making up a substantial percentage of monthly household expenditure in informal settlements in Nairobi [57], it is possible that economic fallout from COVID-19 community lockdowns will cause households that previously cooked with LPG to revert back to polluting cooking fuels (‘reverse fuel switching’). Instances of reverse fuel switching has been documented prior to the COVID pandemic [83], with a prevalence as high as 35% reported in China [87].

### 2.4 Existing evidence of COVID-19 related impacts in Kenya

In April 2020, the proportion of food insecure individuals increased by 38% in Kenya, with regular consumption of fruits decreasing by about 30% [88]. In five informal settlements in Nairobi, over two-thirds (68%) of respondents indicated they had eaten less during lockdown because they did not have enough money to buy food, and food was described by three quarters of participants as their greatest unmet need [58]. Nearly nine in ten (87%) participants in the study by Quaife et al. reported increases in household expenses and 77% reported increases in food prices. Small commercial food businesses in the informal settlements also indicated they were cooking less during lockdown due to the national curfew imposed by the government, which restricted business hours [89]. Women were twice as likely as men to report cooking more frequently (49% versus 24%) and caring for their children (67% versus 36%) while confined to their home during lockdown [58], highlighting the higher burden of household tasks placed on women in these communities as a result of the pandemic [90].

## 3 Data and methods

This study takes place in Mukuru, one of the largest clusters of informal settlements in Nairobi, situated on nearly 650 acres of land in the industrial area of Nairobi along the Nairobi Ngong river [91]. The settlement, which is home to over 150,000 families, has inadequate sanitation and water facilities, poor solid-waste management and no paved roads. Mukuru consists of three main settlements: kwa Reuben, kwa Njenga and Viwandani. The households in this study were specifically recruited from Mukuru kwa Reuben. The residents of the settlement were placed under a COVID-19 lockdown enforced by the Kenyan national government on March 25, 2020, two weeks after the first recorded case of COVID-19 in the country on March 13, 2020. The national lockdown included suspension of international flights, mandatory quarantine of incoming residents, closure of bars and restrictions on restaurant hours of operation. All schools in Kenya were closed on March 15, 2020 and a national dusk-to-dawn curfew (7 p.m.-5 am) was instituted on April 7, 2020.

This longitudinal study aimed to investigate how food and energy consumption patterns changed during the lockdown, and how these characteristics may have interacted during the economic downturn. A baseline, population-based survey on demographics, socioeconomic factors and cooking fuel use patterns was administered to the main cook (or another member of the household, if unavailable) by community health volunteers via door-to-door sampling from December 2019–March 2020 (before the COVID-19 lockdown). Baseline surveys, which took approximately 40 min to complete, concluded with a question asking respondents if they would agree to be contacted again.

Questions about familiarity with a consumer finance mechanism, pay-as-you-go (PAYG) LPG, were asked to a subset of participants at baseline. These questions were included because PAYG LPG commercial companies operate in the community, enabling consumers to purchase gas credits in small payments using smart meter technology with the aim of helping to relieve the financial barrier to LPG access [89]. The higher initial LPG cylinder and equipment costs are absorbed by the PAYG LPG retailer and are transferred to the customer via an upfront security deposit and surcharge to the LPG fuel cost [41]. A sensitivity analysis was conducted using chi-squared tests of independence to compare socioeconomic characteristics between participants asked questions about PAYG LPG and full study sample to check the representativeness of the findings.

Following implementation of a mandatory COVID-19 lockdown in Kenya, a shorter (20 min), telephone-based survey was conducted from April 20–30, 2020 among consenting participants from the baseline survey to document changes in income, household demographics, cooking behaviors and the food environment. To test for potential response bias in the follow-up sample, particularly given the challenges of contacting participants during COVID-19 lockdown, another sensitivity analysis was conducted to compare demographics among the baseline and follow-up sample using chi-squared tests of independence. Both surveys were conducted via smartphones, with data securely transferred to an online storage system using Mobenzi Researcher, a digital platform that has been used successfully in previous health monitoring studies [92].

Descriptive statistics of associations between shifts in cooking patterns, primary cooking fuel used, dietary behaviors and demographics that occurred during lockdown are presented. Additionally, the association between annual per capita LPG consumption (kilograms/capita/year) prior to lockdown with various household socioeconomic and LPG supply-side factors is examined. Annual per capita LPG consumption was a derived variable obtained from two different survey questions: (1) multiplying the self-reported average number of annual refills by the cylinder size used by the household and dividing by the family size; (2) by dividing one year by the self-reported duration that a cylinder typically lasts in the household (to obtain average number of annual refills), multiplying this by the cylinder size used by the household and dividing by the family size. As information about family size was only collected during follow-up, the LPG consumption analysis was limited to the subset (n = 70) of households using LPG that were surveyed during COVID-19 lockdown. With the number of household members potentially shifting during lockdown, a sensitivity analysis examined LPG consumption among only households reporting no change in the number of residents during lockdown. All data analysis was conducted in R version 3.5.1 [93].

Ethical approval was obtained from the University of Liverpool in the United Kingdom, Amref Health Africa and the National Commission For Science, Technology & Innovation (NACOSTI) in Nairobi, Kenya. Informed consent was obtained from all participants prior to conducting the study.

## 4 Results and discussion

### 4.1 Demographics and Socioeconomic Characteristics

A total of 474 randomly selected participants living in Mukuru kwa Reuben completed the baseline survey administered before COVID-19 lockdown, of whom 88% (n = 419) were the main cook of the household. The mean age was 30 years old, and 70% of respondents were female. Two-thirds of respondents had a monthly household income less than or equal to 15,000 Kenyan Shilling (Ksh) (~$140 USD). Nearly all (98%) of respondents reported not having enough income to meet their weekly required spending, and almost half (43%) of households reported seasonal fluctuations in their household income. Nine out of ten households comprised one or two rooms (Table 1). While three-quarters (77%) of respondents reported having access to drinking water in their home, nearly all (97%) used a communal standpipe as their main water source.

**Table 1:** Demographics of baseline sample in Mukuru kwa Reuben.

Characteristic	Baseline Sample (n = 474)
Age (Mean (SD))	30.0 (8.5)
**Gender**	
Female	332 (70%)
**Highest Level of Schooling**	
Primary	122 (25%)
Secondary	162 (60%)
College/university	115 (15%)
**Monthly Household income (Ksh)**	
5000 or less	42 (9%)
5001–15,000	270 (57%)
15,001–25,000	93 (20%)
25,000 or greater	11 (2%)
Don't know/Won't answer	55 (12%)
**Enough money available for weekly required spending**	
Enough	11 (2%)
Not quite enough	249 (53%)
Definitely not enough	211 (45%)
**Household income changes with seasons**	
Yes	201 (43%)
**Occupation (head of household)**	
Day laborer	158 (33%)
Business/government employee	155 (33%)
Business owner	97 (21%)
Unemployed	52 (11%)
Farmer/homemaker	7 (2%)
**Marital Status**	
Married/cohabiting	265 (56%)
Single	192 (41%)
Divorced/widowed	14 (3%)
**Household size (number of rooms)**	
1	128 (27%)
2	291 (62%)
3 +	52 (11%)
**Access to drinking water in home**	
Yes	363 (77%)
**Main water source**	
Communal standpipe	452 (97%)
Pipe in home	15 (2%)
Well or collect from river	3 (1%)

### 4.2 Characterizing the cooking environment at baseline

**Fig. 1:**
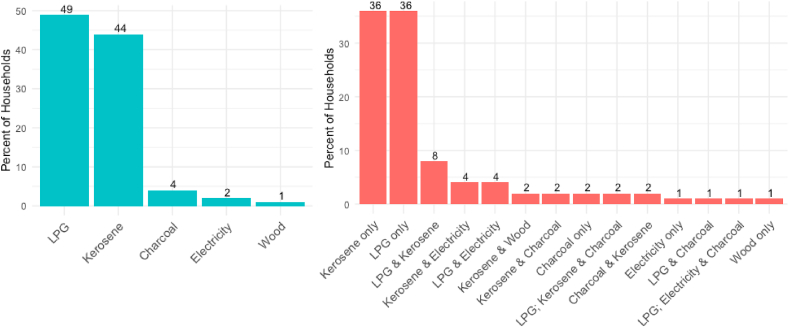
(left). Baseline prevalence of primary fuels in Mukuru kwa Reuben informal urban settlement. (right) Baseline prevalence of primary, secondary and tertiary cooking fuel combinations. Fuels listed in order of primary, secondary and tertiary usage.

Half (49%; n = 232) of baseline survey respondents used LPG as their primary cooking fuel, 44% (n = 207) used kerosene and the remaining households used charcoal/charcoal briquettes (4%; n = 15), electricity (2%; n = 7) or wood (1%; n = 4) (Fig. 1-left). A quarter (26%) of households stacked two or more cooking fuels, with the most common fuel combination being LPG (primary fuel) and kerosene (secondary fuel) (8% of households; n = 38) (Fig. 1-right).

Nine in ten (n = 427) families cooked inside the home in a single room, suggesting minimal levels of ventilation in study households. On a monthly basis, the median cost of LPG (850 ks h) was about half that of polluting cooking fuels sold in the community (1500–1800 ks h). However, the median cost of an LPG cylinder refill (900 ks h) (which 95% of households purchased on a monthly or multi-monthly basis) was over ten times higher than the median cost of polluting fuels (35–70 ks h), which two-thirds of households purchased daily (Table 2). The cost of a single cooking event was much cheaper when using LPG (11 ks h/event), compared with kerosene (17.5 ks h/event) and charcoal (35.0 ks h/event). Approximately 20–25% of participants using the two most common cooking fuels in the community, LPG and kerosene, indicated that the fuel was unavailable for purchase at least four times per year.

**Table 2:** Cooking characteristics by primary cooking fuel type in Mukuru kwa Reuben.

Characteristic	Primary Fuel Type					
	Overall (n = 474)	LPG (n = 232)	Kerosene (n = 207)	Charcoal (n = 15)	Electricity (n = 7)	Wood (n = 4)
Cooking Location						
In main house: no separate room	427 (90%)	196 (84%)	198 (95%)	14 (93%)	7 (100%)	4 (100%)
In main house: separate room	42 (9%)	34 (15%)	6 (3%)	1 (7%)	0	0
Outside of main house: separate room	5 (1%)	2 (1%)	3 (2%)	0	0	0
**Primary Fuel: Weekday Daily Cooking Hours (Median (IQR))**	3.0 (2.0,4,0)	2.9 (2.0, 3.8)	3.0 (2.0, 5.0)	3.5 (2.0, 4.5)	3.0 (2.8, 3.5)	4.0 (2.0, 6.5)
**Primary Fuel: Cooking Events Per Week (Median (IQR))**	18 (7, 21)	18 (7, 21)	18 (7, 21)	12 (7, 19)	21 (1.3)	19 (17, 20)
**Secondary Fuel: Cooking Events Per Week (Median (IQR))**	2 (0, 5)	1 (0, 5)	2 (0, 5)	3 (2, 4)	0 (0, 1)	5 (5, 5)
**Single fuel purchase (KSh) (Median (IQR))**	700 (60, 900)	900(800,1050)	65 (50, 372)	70 (45, 205)	60 (55, 575)	35 (23, 46)
**Monthly Fuel Expenditure (KSh) (Median (IQR))**	1000 (800, 1500)	850 (700, 1100)	1500 (900, 1880)	1800 (1475, 2600)	1500 (625, 1800)	1800 (1400, 1900)
**Fuel cost Per Cooking Event**^a^**(KSh) (Median (IQR))**	20.5 (14.5, 26.1)	11.0 (9.1, 14.3)	17.5 (10.5, 21.9)	35.0 (28.7, 50.6)	16.7 (6.9, 20.0)	22.1 (17.2, 23.3)
**Frequency of fuel purchases**						
Daily	160 (34%)	5 (2%)	137 (66%)	10 (67%)	4 (57%)	4 (100%)
2–14 days	47 (10%)	6 (3%)	39 (19%)	0	2 (28%)	0
Monthly	217 (46%)	181 (78%)	30 (14%)	5 (33%)	1 (14%)	0
2–4 months	41 (9%)	40 (17%)	1 (1%)	0	0	0
**Fuel Availability**						
Always available	194 (42%)	92 (41%)	97 (47%)	3 (20%)	1 (14%)	1 (25%)
Unavailable < 4 times a year	140 (30%)	74 (32%)	52 (25%)	10 (66%)	3 (43%)	1 (25%)
Unavailable 4–12 times a year	59 (13%)	31 (14%)	24 (12%)	1 (7%)	2 (29%)	1 (25%)
Unavailable more than once a month	64 (14%)	28 (13%)	33 (16%)	1 (7%)	1 (14%)	1 (25%)
**Fuel obtained for free**						
Yes	9 (2%)	4 (2%)	4 (2%)	0	0	1 (25%)
**Lighting source(s)**						
Electricity (inc. solar panels) + candle	227 (48%)	118 (51%)	97 (47%)	5 (33%)	7 (100%)	0
Electricity (inc. solar panels) only	50 (11%)	25 (11%)	19 (9%)	3 (20%)	0	0
Electricity(inc. solar panels) + candle + kerosene lamp	33 (7%)	26 (11%)	6 (3%)	1 (7%)	0	0
Electricity(inc. solar panels) + candle + oil/gasoline/LPG lamp	32 (7%)	5 (2%)	26 (13%)	1 (7%)	0	0
Electricity(inc. solar panels) + kerosene lamp	29 (6%)	15 (6%)	14 (6%)	0	0	0
Kerosene lamp	16 (3%)	3 (1%)	11 (5%)	3 (20%)	0	4 (100%)
Electricity (inc. solar panels) + flashlight/lantern or torch	14 (3%)	8 (3%)	6 (3%)	0	0	0
**Household heating**						
No heating	450 (95%)	227 (98%)	193 (93%)	15 (100%)	7 (100%)	2 (50%)

Note: some variables may not add up to 100%; categories were condensed for brevity.

a Median fuel cost per cooking event is a derived variable of fuel cost per month converted into a weekly cost and multiplied by number of cooking events/week.

Approximately 95% (n = 445) of study households used electricity for lighting, but only 11% used it exclusively (Table 2), demonstrating the challenges of solar/grid electricity serving as a reliable energy source for cooking and other more energy intensive activities in this community [94]. Five percent (n = 29) of study households without access to electricity for lighting mostly used kerosene lamps (n = 19; 3% overall prevalence). Nearly half of electrified households used candles in times of power cuts, while other households resorted to kerosene lamps (13%), oil/gasoline/LPG lamps (7%) or flashlights/lanterns/torches (3%). Only 5% of sampled households heat their homes, with most (81%) using a heating fuel for four months or less per year.

#### 4.2.1 Baseline perceptions about cooking with liquefied petroleum gas

**Fig. 2:**
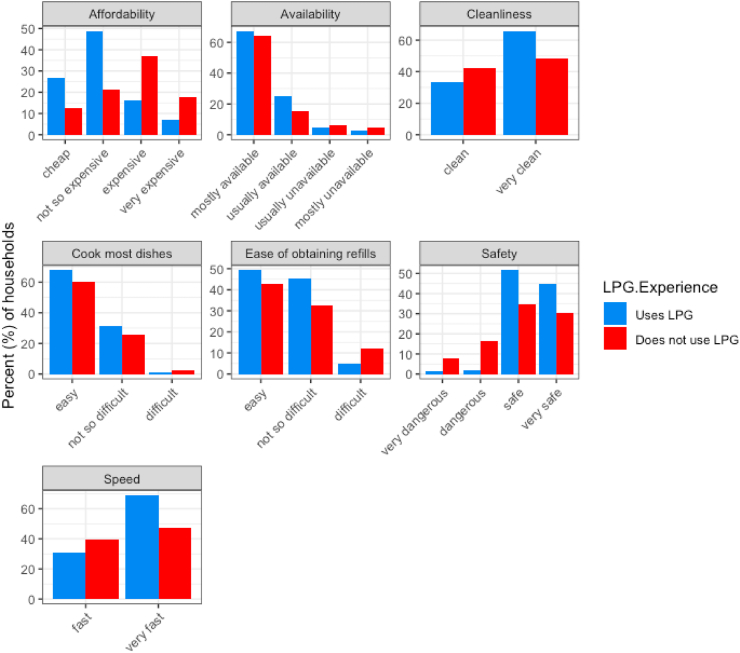
Participants' perceptions about cooking with liquefied petroleum gas (n = 471).

Perceptions about using LPG for cooking were compared between households currently cooking with LPG as a primary or secondary fuel (n = 254) and households cooking with only electricity, kerosene or biomass (charcoal, wood) (n = 217). Over half of households not cooking with LPG believed that cylinder refills were too expensive (Fig. 2). Households cooking with LPG were more likely than those not using the fuel to report LPG as being ‘very clean’, ‘very fast’ and refills being easily obtainable (Fig. 2). Participants using LPG in this study cooked for an hour less (median: 3 h/day) than those cooking with wood (median: 4 h/day) (Table 2). Perceived time savings has previously been stated as an advantage of LPG among this population [89].

No significant differences were reported between households cooking primarily with LPG or biomass in terms of its availability and ability to cook most dishes. Thus, the supply of LPG and its ability to prepare the local cuisine are potentially less important barriers in this community than affordability and convenience of obtaining an LPG cylinder refill. However, as approximately one-third of respondents did not find it easy to cook most dishes with LPG (Fig. 2), fuel stacking may continue to occur among a significant proportion of households if LPG cannot meet all of their cooking needs.

One quarter of households not cooking with LPG had concerns about the safety of LPG (Fig. 2). Safety concerns associated with use of LPG have been cited as a barrier in another informal settlement in Nairobi [82].

Over half (59%; n = 127) of the 217 households not currently using LPG had previous experience of cooking with the fuel. Amongst these, 91% (n = 115) expressed interest in using LPG in the future. Among participants not currently using LPG, the average price at which the LPG stove and equipment would be considered affordable was 5240 ks h ($47.57 USD) (SD: 2650 ks h ($24.06 USD)). The female household head would decide if the family would switch to LPG for cooking in 43% of these households.

**Fig. 3:**
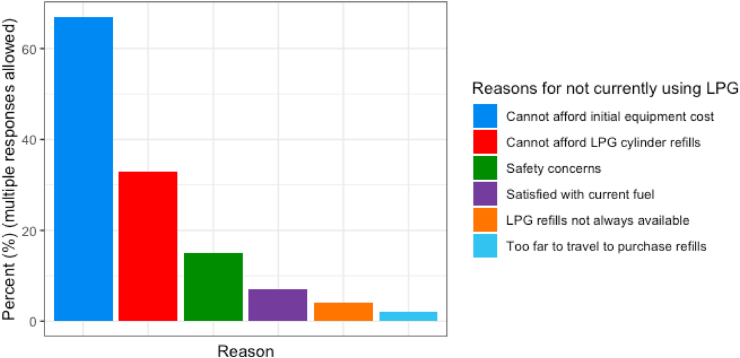
Reasons for households not cooking with LPG at baseline (n = 127).

The upfront cost of the stove and cylinder (67%) and refills (33%) were the most common barriers reported by those currently not cooking with LPG (Fig. 3). An identical analysis conducted in Eldoret, a peri-urban community in western Kenya, showed similar results with nearly 75% of participants expressing that the high cost of the LPG stove and equipment was their main barrier to use [95]. Concerns about the safety of using LPG due to fire hazards from gas leaks prevented a slightly higher proportion of the population in Mukuru kwa Reuben (15%) from deciding to use LPG than in Eldoret (6%), possibly due to recent LPG cylinder explosions that occurred in Mukuru kwa Reuben, attributed to faulty values and illegally refilled cylinders [96].

#### 4.2.2 Liquefied petroleum gas usage characteristics before lockdown

Three quarters (73%; n = 186) of households primarily cooking with LPG used their gas stove every day (Table 3). Nearly all (98%) households using LPG owned only one cylinder, with the most common (85%; n = 222) cylinder size being 6 kg (kg). A 6 kg cylinder lasted participants about one and a half months on average between refills, equating to an average of eight cylinder refills per year (Table 3). One-third (33%) of participants reported having to pay 1–100 ks h to travel to the LPG retailer where they would obtain their refill.

**Table 3:** Characteristics of households cooking with LPG at baseline (n = 256).

No. of days using LPG during the last week	N (%)
0–4	27 (11%)
5–6	42 (16%)
7	186 (73%)
**Size of LPG cylinder**	a
3 kg	14 (5%)
6 kg	222 (85%)
13 kg	25 (10%)
**Annual refills among 3 kg cylinder owners (Mean (SD))**^a^	5.8 (2.9)
**Annual refills among 6 kg cylinder owners (Mean (SD))**^a^	8.0 (3.0)
**Annual refills among 13 kg cylinder owners (Mean (SD))**^a^	5.5 (3.3)
**Number of burners**	
1 burner on top of cylinder	197 (78%)
1 burner separate from cylinder	19 (8%)
2 burners	36 (14%)
**Years since last LPG stove purchase**	
< 1	79 (31%)
1–2	129 (51%)
2–5	38 (15%)
5 +	8 (3%)
**Mode of transportation for LPG refills**	
On foot	183 (73%)
By scooter/motorbike/bicycle	67 (26%)
Home delivery	3 (1%)
**Travel cost to reach LPG vendor (Ksh)**	
No cost	172 (67%)
20–50	50 (20%)
51–100	22 (9%)
> 100	9 (4%)
**Cost of LPG home delivery in your area (Ksh) (n = 66)**	
50	46 (70%)
100	13 (20%)
150	7 (10%)

a Number of annual refills calculated by dividing one year by average duration of a cylinder.

Using self-reported data on average length of time until the gas is depleted in a typical cylinder, average annual per capita LPG consumption in the community was 48 kg/capita/year (range: 3.6, 72.0). Although self-reported data LPG consumption values should be interpreted carefully due to potential reporting bias [97], there were not substantial differences between the two different survey questions from which LPG per capita consumption was derived (number of annual cylinder refills and average amount of time until all gas in a cylinder is used) (see Supplemental Information; Fig. S1). Annual per capita LPG consumption was reported among households participating in follow up surveys due to omission of questions on family size in the baseline survey; a sensitively analysis conducted among a subset of 57 of the 70 households that did not indicate a change in number of residents during lockdown revealed no significant changes in LPG consumption levels (see Supplemental Information; Fig. S1).

**Fig. 4:**
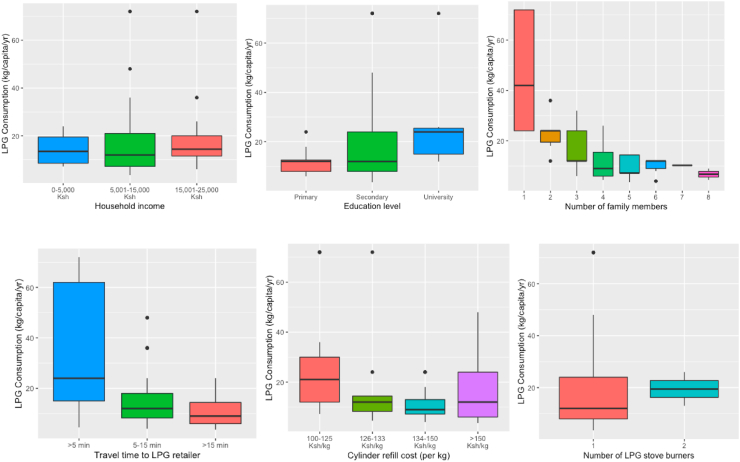
Relationship between household socioeconomic factors, supply-side factors and annual per capita LPG consumption prior to lockdown.

Supply-side characteristics, including decreasing travel time to the nearest LPG retailer and lower cylinder refill cost, as well as owning a double-burner stove and having a smaller family were more positively associated with increasing annual per capita LPG consumption than increasing household income (Fig. 4). While only bivariable analyses are presented due to insufficient power to build robust multivariable models, supply-related factors were similarly found to be more highly predictive of LPG consumption than household income in a modeling study of over 5500 households in peri-urban communities of Kenya, Ghana and Cameroon [95]. Taken together, these findings provide empirical evidence that access to affordable LPG refills, double-burner LPG stoves and living in close proximity (<5 min travel time in an urban context) to an LPG supplier are important supply-related considerations for increasing LPG consumption in Nairobi and in peri-urban communities. The importance of these factors may reflect the high value placed on time savings among LPG users; individuals in the community have indicated that a double burner LPG stove is advantageous over a kerosene stove because of the ability to cook ugali and vegetables simultaneously [89] and customers may value the convenience of living nearby an LPG retailer.

#### 4.2.3 Pay-as-you-go liquefied petroleum gas

Due to the existence of two commercial companies offering pay-as-you-go (PAYG) LPG to customers in Mukuru kwa Reuben [41], a subset of participants (n = 107) were questioned about their perceptions of PAYG smart-meter technology. This subset was similar in demographic profile to the full baseline sample (see Supplemental Information; Table S1). Half of participants (n = 52) expressed interest in learning more about PAYG LPG (Table 4). Interest was three times as high among those primarily cooking with kerosene (74%) compared with existing LPG users (27%), and 30% of households using kerosene reported an intention to register with a PAYG LPG company (Table 4). Respondents that expressed interest in registering with a PAYG LPG commercial company operating in the community, had most commonly been introduced to the technology via word of mouth (59%; n = 30) (Table 4). Community health volunteers, who have a high level of trust in the community [98], were responsible for introducing one quarter (24%; n = 12) of households.

**Table 4:** Interest in pay-as-you-go (PAYG) LPG smart-meter technology.

Characteristic	Overall (n = 107)	LPG (n = 52)	Kerosene (n = 43)	Charcoal (n = 6)	Electricity (n = 6)
Interest in learning more about PAYG LPG					
Yes	52 (49%)	14 (27%)	32 (74%)	2 (33%)	4 (67%)
**Most attractive feature of PAYG LPG**					
Ability to pay for gas in small increments/via mobile money	41 (38%)	15 (29%)	21 (49%)	2 (33%)	2 (33%)
Increased safety	26 (24%)	14 (27%)	8 (19%)	2 (33%)	2 (33%)
Customer service/advanced Technology	17 (16%)	7 (13%)	7 (16%)	2 (33%)	1 (17%)
Ability to track gas during each Meal	7 (7%)	6 (12%)	1 (2%)	0	0
Eliminates need to travel for refills	4 (4%)	3 (6%)	1 (2%)	0	0
Not heard of PAYG LPG	8 (8%)	4 (8%)	4 (9%)	0	0
**Desire to register with a local PAYG LPG commercial company**					
Intend to register in the future	23 (22%)	8 (15%)	13 (30%)	0	2 (33%)
Not interested in registering	23 (22%)	17 (33%)	4 (9%)	1 (17%)	1 (17%)
Already registered/received Equipment	5 (4%)	1 (2%)	2 (5%)	1 (17%)	1 (17%)
Not heard of the PAYG company but interested in learning more	22 (21%)	5 (10%)	15 (35%)	1 (17%)	2 (33%)
Not heard of the PAYG company and not interested in learning more	33 (31%)	21 (40%)	9 (21%)	3 (50%)	0
**Method of learning about PAYG LPG commercial company (N = 51)**					
Word of mouth	30 (59%)	12 (50%)	12 (63%)	2 (100%)	2 (50%)
From community health volunteer	12 (24%)	8 (33%)	2 (11%)	0	2 (50%)
Door to door advertising	9 (17%)	4 (17%)	5 (26%)	0	0

The most attractive feature of PAYG LPG listed by participants was ability to pay for gas in small amounts (37%). The proportion of participants cooking with kerosene that found the ability to purchase gas in small amounts to be the most attractive feature of PAYG LPG (49%) was nearly twice as much as that of existing LPG users (29%).

A quarter of participants cited increased safety (27%) of PAYG LPG as its main advantage. A recent study that included interviews with customers of a PAYG LPG company residing in the same community revealed that they felt safer knowing that the company's customer support team could detect gas leaks and remotely turn off the gas, thereby minimizing fire hazards [89]. PAYG commercial companies also follow standard operating procedures with the cylinders they deliver directly to households, thereby preventing illegal cylinder refills. Customers in the community also valued the time savings (from use of a double burner versus single burner stove), cylinder delivery and user-friendliness of PAYG LPG [89].

### 4.3 Follow up surveys during COVID-19 lockdown

The sampling frame for follow up telephonic surveys administered during the COVID-19 lockdown in April 2020, consisted of 60% (n = 285) of baseline respondents who consented to be contacted again. The final analytic sample consisted of 194 out of 285 participants (41% of original baseline survey respondents). A lower-than-expected follow-up was primarily due to participants’ mobile phone being switched off (likely because of inability to pay mobile phone bills during the lockdown, and lack of power connectivity for charging among those who had travelled to rural villages). No statistically significant difference in socioeconomic characteristics was observed between the 474 baseline and 194 follow-up participants (Table 5).

**Table 5:** Comparison of baseline and follow up sample demographics.

Characteristic	Baseline Sample (n = 474)	Follow Up Sample (n = 191)	Test statistic (χ^2^ or *t*-test) p-value
Age (Mean (SD))	30.0 (8.5)	31.5 (8.6)	1.39 p = 0.17
**Gender**			
Female	332 (70%)	141 (74%)	0.77 p = 0.37
**Highest Level of Education**			
Primary	122 (25%)	51 (27%)	1.75 p = 0.41
Secondary	278 (60%)	119 (62%)	
College/university	70 (15%)	21 (11%)	
**Monthly Household Income (Ksh)**			
5000 or less	42 (9%)	20 (10%)	6.53 p = 0.16
5001–15,000	270 (57%)	118 (61%)	
15,001–25,000	93 (20%)	26 (14%)	
25,000 or greater	11 (2%)	1 (1%)	
Don't know/Won't answer	55 (12%)	26 (14%)	
**Occupation (head of household)**			
Day laborer	158 (33%)	66 (34%)	1.65 p = 0.81
Business/government employee	155 (33%)	58 (30%)	
Business owner	97 (21%)	42 (21%)	
Unemployed	52 (11%)	27 (14%)	
Farmer/homemaker	7 (2%)	2 (1%)	
**Marital Status**			
Married/cohabiting	265 (56%)	115 (60%)	0.87 p = 0.65
Single	192 (41%)	71 (37%)	
Divorced/widowed	14 (3%)	5 (3%)	
**Household Size (number of rooms)**			
1	128 (27%)	37 (19%)	4.44 p = 0.11
2	291 (62%)	130 (68%)	
3 +	52 (11%)	24 (13%)	
**Baseline Primary Cooking Fuel**			
LPG	232 (49%)	70 (37%)	11.52 p = 0.02^a^
Kerosene	207 (44%)	111 (58%)	
Charcoal	15 (4%)	7 (4%)	
Electricity	7 (2%)	1 (1%)	
Wood	4 (1%)	2 (1%)	

a = statistically significant at alpha = 0.05 level.

### 4.4 Effects of COVID-19 lockdown on income and food security

Nearly all participants (95%; n = 181) reported a decline in household income during lockdown, with one third (34%; n = 65) indicating no income coming into their household (Table 6). Nearly nine in ten households (88%, n = 168) reported being food insecure due to insufficient

**Table 6:** Effect of COVID-19 lockdown on food security in Mukuru kwa Rueben informal urban settlement (n = 194).

Characteristic	Number of households (%)
Change in income	
No income coming into household	65 (34%)
Less income (not enough)	105 (54%)
Less income (but enough)	13 (7%)
No change	11 (5%)
**Have enough food to feed family**	
No	177 (91%)
**Reasons why food insecure (select all) (n = 177)**	
Not enough income	177 (100%)
More people to feed in the household	8 (5%)
Cannot travel to local shop/market	4 (2%)
**Change in type of food cooked**	
Yes	101 (52%)
**Food source during lockdown**	
Local shop/market (same as before lockdown)	133 (69%)
Local shop/market (different location than before lockdown)	37 (19%)
Friends/family/source from home (same as before lockdown)	8 (4%)
Friends/family/source from home (different than before lockdown)	16 (8%)
Number of household residents (Mean (SD))	3.6 (1.9)
**Change in number of household residents**	
More residents	14 (8%)
Fewer residents	28 (16%)
Same	134 (76%)
**Change in cooking frequency**	
Much less frequent	23 (12%)
Less frequent	80 (41%)
No change	80 (41%)
More frequent	11 (6%)

income. This percentage is 14% higher than that from another study conducted in multiple informal settlements in Nairobi, in which 74% percent of households indicated skipping or eating less due to lower income [58]. While 77% of people in the study by Quaife et al. reported increases in food prices, the World Food Programme did not identify substantial food price increases (less than 5%) in Kenya in April 2020 [99] and the Food and Agricultural Organization (FAO) reported that food prices have not shown significant increases due to COVID-19 [45]. The report by FAO stated that, while food availability may have declined in the medium term (3–6 months) due to import delays, the Kenyan Ministry of Agriculture, Livestock, Fisheries and Cooperatives listed foodstuff and farm inputs as essential goods to facilitate imports into the country to minimize disruptions to the supply chain. The International Monetary Fund found that, despite 15% of food imports into Kenya affected by export bans [100], lower incomes due to COVID-19 were likely a more important driver of food insecurity than supply-side impacts [101]. FAO further states that prices of key staple foods in Kenya had increased before COVID-19 lockdowns took effect, with the cost of food rising nearly 12% percent in March of 2020 over the same month in the previous year.

**Fig. 5:**
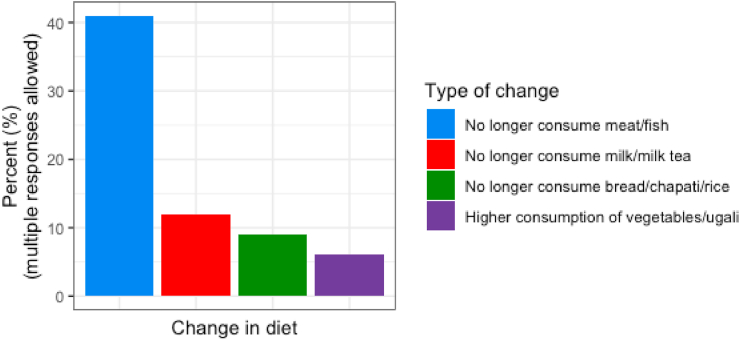
Changes in dietary behaviors reported in Mukuru kwa Reuben as a result of lockdown (open ended question) (n = 101).

Half (52%) of participants indicated changing the type of food they cooked during lockdown. Common dietary changes were reductions in meat/fish, milk/milk tea and bread/chapati, with higher consumption of vegetables (Fig. 5). This may have increased the likelihood of protein deficiency in the population [102], with the majority of Kenyans already protein deficient prior to COVID-19 partially due to insufficient diversification of food consumption and the high cost of meat and fish [103].

**Fig. 6:**
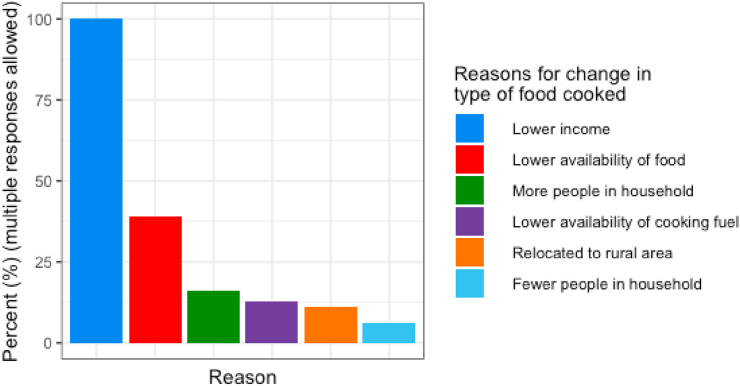
Reasons for a change in type of food cooked during lockdown (multiple options allowed) (n = 101).

In addition to insufficient income, lower food availability (39%) was cited as a main reason for participants altering their dietary behavior (Fig. 6). One quarter (27%) of households switched their main food vendor/retailer during the lockdown, with 8% (n = 16) of households resorting to farming and livestock as their new primary food source.

**Fig. 7:**
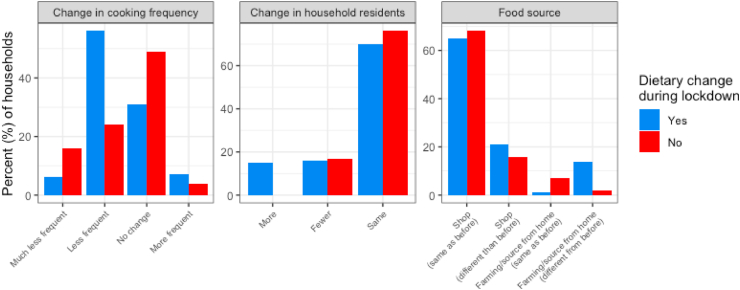
Factors affecting dietary changes during lockdown (n = 101).

Over half of households (53%; n = 103) cooked less frequently during the lockdown (Table 6). Participants changing the foods they cooked were twice as likely to cook less frequently (56%; n = 56) than those not changing the types of foods they cooked (26%; n = 24) (Fig. 7). Nearly all (88%; n = 14) households that shifted their main source of food to farming and livestock reported changing their diet (Fig. 7). Additionally, all 15 households that had more residents during lockdown reported changing the foods they cooked. Thus, shifts in dietary consumption were associated with not only variations in the food environment, but changing household demographics and cooking patterns. Households obtaining their food from markets were more likely to change their food consumption than those sourcing from farmland and livestock (Fig. 7), which matches with findings from a national survey conducted in Kenya [88].

### 4.5 Effects of COVID-19 lockdown on cooking fuel use

**Fig. 8:**
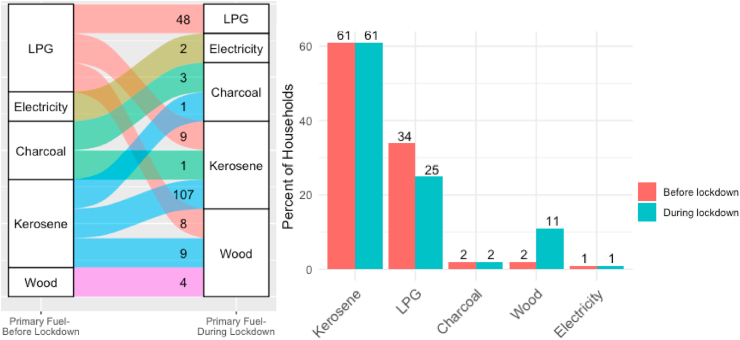
(left). Number of households switching between different primary fuel types during lockdown. (right) Prevalence (%) of primary fuel types in Mukuru kwa Rueben informal urban settlement before and during lockdown (n = 194).

Primary cooking fuel switching occurred among 14% (n = 27) of households in response to the lockdown, with LPG users (26%) being three times more likely to switch their primary cooking fuel than kerosene users (8%). Previous LPG users switched to kerosene (n = 9) or wood (n = 8), and nine households previously using kerosene switched to wood (Fig. 8-left). The prevalence of wood being used as a cooking fuel in the community increased by 9% (2%–11%) during lockdown, in conjunction with a 9% decline in LPG use for cooking (34%–25%) (Fig. 8-right).

Although some families that switched from LPG to kerosene or wood indicated cooking less frequently during lockdown (Table 7), the length of exposure to household air pollution (HAP) from burning these fuels may still increase, due to a longer time spent indoors in a cramped area (89% of households had less than three rooms) and a 1-h longer median cooking time reporting among households using wood rather than LPG (Table 2). Research that examined cooking fuel usage in the peri-urban community of Eldoret in western Kenya also showed a considerable increase in use of wood and charcoal for cooking and a simultaneous decline in LPG during COVID-19 lockdown [89]. It is possible that elevated HAP emissions from increased use of polluting fuels in the population will contribute to a rise in HAP concentrations at a community level [104,105] as smoke from biomass combustion can infiltrate neighboring homes [106], especially with tightly-packed housing in an informal settlement [49,107].

**Table 7:** Demographic and dietary behaviors by primary cooking fuels used before and during lockdown (households using LPG or kerosene prior to lockdown only).

	Change in primary cooking fuel during lockdown						
	LPG to wood (n-8)	LPG to kerosene (n = 9)	No change (always LPG) (n = 48)	Kerosene to wood (n = 9)	No change (always kerosene) (n = 107)	Any cooking fuel change (n = 26)	No cooking fuel change (n = 158)
Change in number of household members							
More	4 (50%)	0	2 (4%)	2 (29%)	7 (6%)	6 (23%)	9 (6%)
Fewer	1 (13%)	2 (22%)	7 (15%)	0	19 (17%)	3 (12%)	26 (17%)
Same	3 (38%)	7 (78%)	39 (81%)	7 (71%)	81 (74%)	17 (65%)	128 (82%)
**Change in cooking frequency**							
Much less frequent	1 (12%)	0	8 (17%)	0	10 (9%)	1 (4%)	18 (12%)
Less frequent	2 (25%)	8 (89%)	17 (35%)	4 (44%)	47 (43%)	14 (54%)	64 (41%)
No change	4 (50%)	1 (11)	20 (42%)	3 (33%)	47 (43%)	8 (31%)	67 (43%)
More frequent	1 (12%)	0	3 (6%)	2 (22%)	3 (3%)	3 (11%)	6 (4%)
**Change in foods cooked during lockdown**							
Yes	7 (88%)	6 (67%)	14 (29%)	9 (100%)	58 (54%)	22 (85%)	74 (46%)
No	1 (12%)	3 (33%)	34 (71%)	0	49 (46%)	4 (15%)	84 (54%)

### 4.6 Factors associated with cooking fuel switching during lockdown

Among 15 participants that indicated having more residents living with them during lockdown (Table 7), 40% (n = 6) switched from LPG (4) or kerosene (2) to wood. In comparison, only 12% (n = 17) of the 145 households with the same number of members during lockdown switched their primary cooking fuel. Thus, some households may not have only switched to gathering wood because it was freely available, but also because they could more easily prepare larger meals to accommodate having more people to feed (Table 7) [80].

Additionally, the 12% (n = 17) of households with same number of members during lockdown that decided to switch their primary cooking fuel may have had to prepare additional meals for their children who would otherwise have been provided lunch at school [89]. The World Food Programme predicts that at the peak of lockdown in April 2020, 360 million children were not receiving school meals [108], signaling that increased home cooking occurred on a large scale.

While the sample size in this study is too small to draw definitive conclusions, switching to firewood may have been a strategy used by participants to eliminate purchases of costly LPG refills so they could direct a higher proportion of their income toward food purchases [34]. Given that household energy makes up a significant portion of monthly household expenditure (up to 20%) in Mukuru kwa Reuben [57], freely available firewood may have been viewed as a desirable option to cut a significant proportion of household expenditures. As LPG has been shown to provide households more diverse diets than those cooking with biomass in some instances [35], minimizing the costs of LPG refills (e.g. via fuel subsidies) may help households maintain a more balanced diet that can protect against malnourishment [34].

As nearly all (89%) households switching from LPG to kerosene reported cooking ‘less frequently’ (Table 7), it is possible that this fuel switch occurred because kerosene fuel could be purchased in daily increments. In contrast, nearly all households (n = 18) that reported cooking ‘much less frequently’ did not switch their primary cooking fuel during lockdown (Table 7). Thus, a participants' decision to stop using LPG during a period of financial strain may have partially reflected whether priority was given to continued use of their clean cooking fuel or the capacity to prepare larger meals (e.g. using firewood) that would be sufficient food to feed their family. As LPG is viewed by many in the community as a status symbol [82], whether or not cooking with LPG was viewed by participants as a privilege or a necessity may have played a role in household energy decisions during lockdown.

The choice to change cooking fuels can also be more nuanced, as cooking decisions are influenced in part by food and fuel availability. Four in five (81%; n = 22) of the 27 households that switched their primary cooking fuel reported preparing different foods during lockdown (Table 7) and nine participants that did not change their primary cooking fuel during lockdown reported changing the food they cooked due to low availability of cooking fuel (Fig. 6). In contrast, less than half (47%; n = 79) of households that cooked with the same fuel during lockdown (n = 167) altered the foods they cooked.

**Fig. 9:**
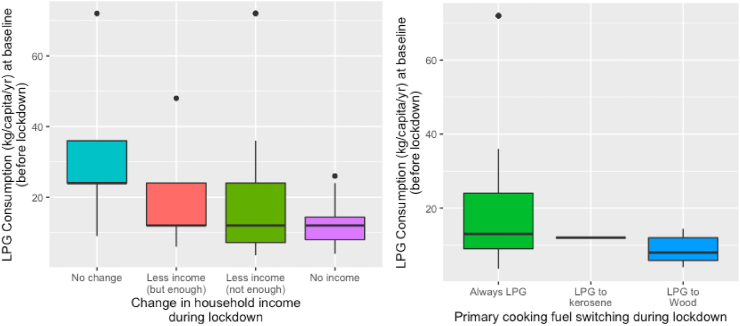
Relationship between changes in income or cooking fuel during COVID-19 lockdown and annual per capita LPG consumption prior to lockdown.

While there was not a significant relationship between household income and LPG consumption at baseline (Fig. 4), an inverse relationship was found between amount of LPG consumed prior to lockdown and the degree of income loss during lockdown (Fig. 9); households reporting having no income during lockdown consumed less LPG per capita annually prior to lockdown than households reporting that their income was unaffected by lockdown. Additionally, households that switched their primary cooking fuel from LPG to wood were consuming nearly half the amount of LPG per capita (mean: 8.0 kg/capita/year) than those continuing to use LPG during lockdown (mean: 15.3 kg/capita/yr) (Fig. 9). This suggests that a negative economic effect of the COVID-19 lockdown may be an exacerbation of existing inequities in terms of clean cooking access and consumption among the urban poor [86]. A recent study in Kenya found that the lowest income households were more vulnerable to income shock, and were more likely to employ food-based coping strategies during COVID-19 lockdown compared to those with moderately higher incomes [88]. Given the evidence showing that lower LPG consumption in the community was associated with a higher cost of LPG cylinder refills (Fig. 4), these findings underline that similar coping strategies may have been applied to cooking fuel decisions during lockdown, with a proportion of households quickly replacing their cooking fuel in response to a decline in income [34].

### 4.7 Change in primary cooking fuel costs during lockdown

**Fig. 10:**
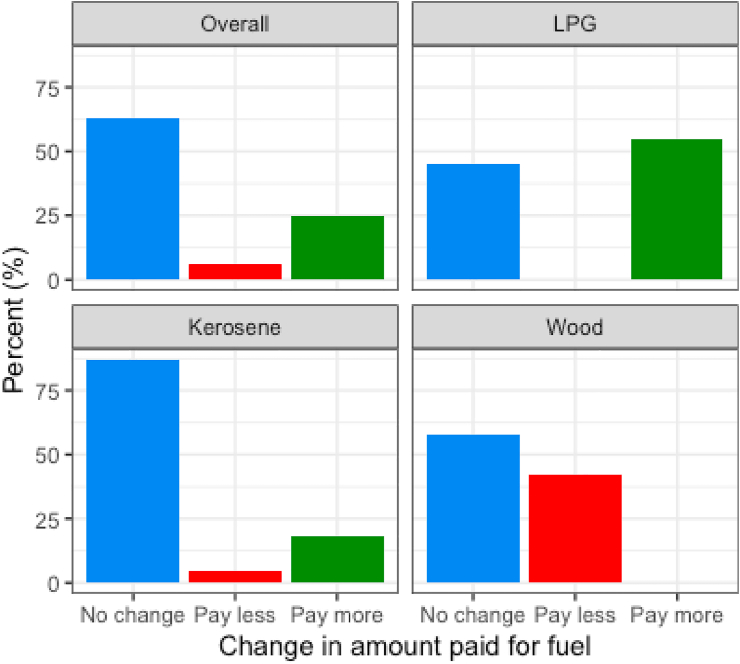
Change in fuel cost (Ksh) during lockdown by primary fuel type (n = 183).

Compounding the clean energy access barriers resulting from lower household income, over one quarter (29%; n = 50) of the 172 households that continued to purchase their cooking fuel during lockdown reported paying a higher price for their primary cooking fuel (Fig. 10). The percent of households using LPG that paid greater than 1000 ks h ($9.10 USD) for cylinder refills increased by 55% during lockdown (4%–59%), compared with an 8% rise (3%–11%) among kerosene users (Table 8). The proportion of households that reported paying less for cooking fuel was highest among those primarily using wood (42%; n = 5). 43% of households reported gathering wood for free during the lockdown.

**Table 8:** Cost of fuel before and during lockdown by primary fuel type (n = 194).

Fuel cost before/during lockdown (Ksh)	Overall (N = 183)		LPG (N = 49)		Kerosene (N = 117)		Wood (N = 21)	
	Before	During	Before	During	Before	During	Before	During
Free	6 (3%)	10 (6%)	0	0	1 (1%)	0	5 (24%)	9 (43%)
1–500	107 (55%)	94 (49%)	3 (6%)	2 (4%)	89 (76%)	76 (65%)	10 (46%)	12 (57%)
501–1000	75 (39%)	46 (24%)	44 (90%)	18 (37%)	23 (20%)	27 (23%)	6 (29%)	0
> 1000	6 (3%)	43 (22%)	2 (4%)	29 (59%)	4 (3%)	13 (11%)	0	0

In the same community, the prevalence of cooking fuel switching among PAYG LPG customers who had the option to pay for LPG incrementally during lockdown (5%) was lower than that among full cylinder LPG users in this study (22%) [89]. Indeed, noticeable shifts in payment patterns among PAYG LPG customers during lockdown were detected, with lower mobile money payments being made more frequently, reflecting lower cash on hand. Taken together, these findings demonstrate that PAYG LPG, and potentially other consumer finance mechanisms (e.g. microfinance, unconditional cash transfers [109]) may help mitigate declines in clean energy access due to higher LPG prices and lower household income resulting from the COVID-19 pandemic.

### 4.8 Strengths and limitations

While this pre-post study had a lower-than-expected follow up rate (41%), primarily due to participants discontinuing their use of mobile phones either due to financial hardship or inability to charge their device, this analysis has the advantage of documenting the immediate effects of a COVID-19 community lockdown in an informal settlement in Nairobi, Kenya. A follow up sample of 194 participants revealed significant linkages between shifting household demographics, income, foods cooked, frequency of cooking and changes in cooking fuels used during COVID-19 community lockdown. While all data is self-reported, the significant socioeconomic shifts provide strong evidence of the negative side-effects of confinement measures on the livelihoods of informal settlers living in Nairobi and provided an opportunity to explore various relationships between food and energy insecurity during an economic downturn.

Despite the narrow geographic coverage of this study, the impacts of COVID-19 lockdown on income and food security identified are aligned with those reported by another study conducted among five informal settlements in Nairobi [58], suggesting that the validity of our results may well extend to the two million residents living in informal settlements in the city. However, as household energy access is a multifactorial issue influenced by fuel availability, price, proximity, family composition, cultural preferences and convenience [3], the effects of the COVID-19 pandemic observed in this study may not hold in other urban or rural areas of SSA, particularly due to a differential approach to lockdown between some countries (e.g. differences in trade and border restrictions influencing food and fuel supply).

## 5 Conclusion

This longitudinal study highlights the downstream impacts of financial strain from a mandatory COVID-19 lockdown on household energy and dietary decisions. Significant linkages between household income, demographics, foods cooked, frequency of cooking and changes in cooking fuel used were found. It is evident that fuel cost and availability influence the type of cooking fuel used, which, in turn, may dictate household dietary behaviors via multiple pathways, including the ability to cook certain types of foods to meet taste preferences [82], capacity to cook larger meals [80] and cooking frequency [34]. It is further shown that COVID-19 may deepen inequities in clean cooking fuel access, with lower LPG consumption at baseline found among households reporting higher income losses during COVID-19 lockdown than households reporting no changes in income between baseline and follow up.

As expanding the LPG market in Kenya and across SSA presents a viable, medium-term pathway to achieving SDG 7 (universal clean energy access), this study uncovers the need for LPG to be affordable while satisfying families’ cooking and dietary needs, which can also help meet SDG 2 (zero hunger). Consumer-tailored options, such as PAYG LPG smart meter technology have the potential to offset these key barriers to clean energy access in urban areas of Kenya, by allowing for smaller payments and the ability to cook multiple meals simultaneously using double-burner stoves. Promotion of multi-burner stoves and subsidies that lower fuel costs can potentially enable larger families to cook with LPG more frequently and minimize fuel stacking or fuel switching during periods of economic downturn.

The strong associations between demographic, dietary and cooking patterns reveal important lessons for African governments and other stakeholders to rethink their strategies for increasing access to clean cooking post-COVID-19 crisis. Policies in the post-COVID-19 era that focus on improving clean fuel affordability, accessibility and compatibility with the cooking needs of families can help promote energy and food security among the urban poor.

## Declaration of competing interest

The authors declare that they have no known competing financial interests or personal relationships that could have appeared to influence the work reported in this paper.

## Data Availability

Survey data collected as part of this study will be made available on a case-by-case basis on request to the corresponding author, with input from the co-authors, subject to compliance with Research Ethics Board restrictions.
