# Supplementary material for: COVID-19 impacts on household energy & food security in a Kenyan informal settlement: The need for integrated approaches to the SDGs
Source: Renew Sustain Energy Rev. 2021 Jul;144:None. doi: 10.1016/j.rser.2021.111018 (PMC8262075; doi:10.1016/j.rser.2021.111018)
Supplement: Multimedia component 1 [file mmc1.docx]

**SUPPLEMENTAL INFORMATION**

**COVID-19 Impacts on Household Energy & Food Security in a Kenyan Informal Settlement: The Need for Integrated Approaches to the SDGs**

Matthew Shupler^1*^, James Mwitari^2^, Arthur Gohole^2^, Rachel Anderson de Cuevas^1^, Elisa Puzzolo^1,3^, Iva Čukić^1^, Emily Nix^1^, Daniel Pope^1^

1. Department of Public Health and Policy, University of Liverpool, Liverpool, United Kingdom

2. School of Public Health, Amref International University, Nairobi, Kenya

3. Global LPG Partnership (GLPGP), 654 Madison Avenue, New York, United States

*Corresponding author: [m.shupler@liverpool.ac.uk](mailto:m.shupler@liverpool.ac.uk)

***LPG Consumption***

Annual per capita LPG consumption was calculated only among households participating in follow up surveys due to omission of data on family size in the baseline survey. Annual per capita LPG consumption was derived in two different ways: using number of annual cylinder refills and average amount of time a cylinder lasts until it runs empty. No substantial differences were found in the distribution of annual per capita consumption using both survey variables (Figure S1-left). A sensitively analysis conducted among a subset of 57 of the 70 households that did not indicate a change in number of residents during lockdown revealed no significant changes in LPG consumption patterns (Figure S1-right).

It is noted that LPG consumption in this study is susceptible to reporting bias. A previous study comparing self-reported LPG fuel consumption (based on number of annual refills) with sales data on number of cylinders purchased in India found that 85% of respondents overreported their LPG consumption [1].


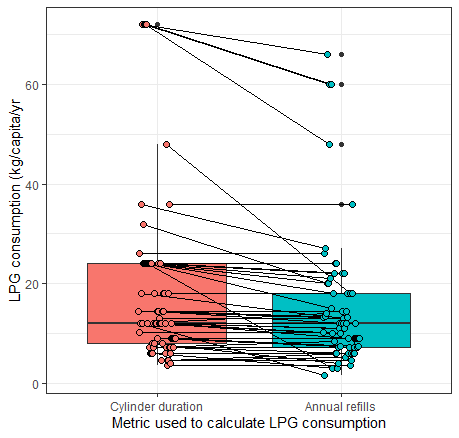

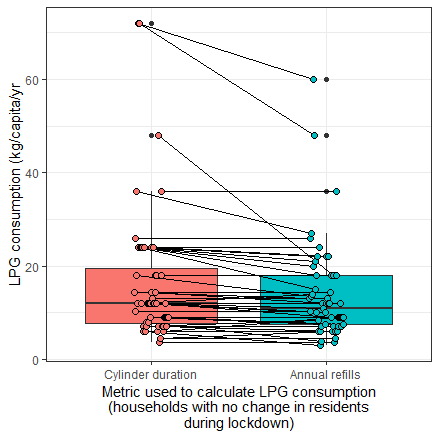


Figure S1. Comparison of LPG per capita consumption using number of annual refills and average duration between refills among all households (n=70; left) and households reporting no changes in number of residents during lockdown (n=57; right)

***Sensitivity Analysis***

A sensitivity analysis was conducted to compare the baseline sample (n=474) to the subset of participants asked questions about pay-as-you-go (PAYG) liquefied petroleum gas (LPG) technology (n=107). Using Pearson’s chi-squared tests (categorical data) and two-sample t-tests (continuous data), participants that were asked additional questions about their perceptions of PAYG LPG had a smaller household size (lower number of rooms) than the overall sample (p=0.01) (Table S1). No other statistically significant differences were found among demographics and socioeconomic characteristics between the two study samples.

**Table S1. Comparison of Baseline and Pay-As-You-Go (PAYG) LPG Sample Demographics**

| Characteristic | Full Baseline Sample (n=474) | PAYG LPG Sample (n=107) | Test statistic  (χ^2^ or t-test)  P-value |
| --- | --- | --- | --- |
| Age (Mean (SD)) | 30.0 (8.5) | 29.2 (9.4) | 0.77  p=0.44 |
| Gender |  |  |  |
| Female | 332 (70%) | 76 (71%) | 0.01  p=0.93 |
| Highest Level of Schooling |  |  |  |
| Primary | 122 (25%) | 26 (24%) | 0.01  P=0.93 |
| Secondary | 162 (60%) | 71 (66%) |  |
| College/university | 115 (15%) | 10 (10%) |  |
| Monthly Household income (Ksh) |  |  |  |
| 5,000 or less | 42 (9%) | 10 (9%) | 3.54  p=0.47 |
| 5,001 – 15,000 | 270 (57%) | 64 (60%) |  |
| 15,001 – 25,000 | 93 (20%) | 24 (22%) |  |
| 25,000 or greater | 11 (2%) | 3 (3%) |  |
| Don’t know/Won’t answer | 55 (12%) | 6 (6%) |  |
| Occupation (head of household) |  |  |  |
| Day laborer | 158 (33%) | 41 (38%) | 3.54  p=0.47 |
| Business employee | 155 (33%) | 38 (35%) |  |
| Business owner | 97 (21%) | 21 (20%)) |  |
| Unemployed | 52 (11%) | 5 (5%) |  |
| Farmer/homemaker | 7 (2%) | 2 (2%) |  |
| Marital Status |  |  |  |
| Married/ cohabiting | 265 (56%) | 68 (63%) | 2.03  p=0.36 |
| Single | 192 (41%) | 37 (35%) |  |
| Divorced/widowed | 14 (3%) | 2 (2%) |  |
| Household size (number of rooms) |  |  |  |
| 1 | 128 (27%) | 39 (36%) | 8.83  p=0.01* |
| 2 | 291 (62%) | 65 (61%) |  |
| 3+ | 52 (11%) | 3 (3%) |  |
| Primary Cooking Fuel |  |  |  |
| LPG | 232 (49%) | 52 (48%) | 9.11  p=0.06 |
| Kerosene | 207 (44%) | 43 (40%) |  |
| Charcoal | 15 (4%) | 6 (6%) |  |
| Electricity | 7 (2%) | 6 (6%) |  |
| Wood | 4 (1%) | 0 |  |

*=statistically significant at alpha=0.05 level

**References**

[1] Kar A, Brauer M, Bailis R, Zerriffi H. The risk of survey bias in self-reports vs. actual consumption of clean cooking fuels. World Development Perspectives 2020;18:100199. https://doi.org/10.1016/j.wdp.2020.100199.
